# Pancreas-sparing tumor resection for peripancreatic paraganglioma: a case series of six patients

**DOI:** 10.1093/jscr/rjae205

**Published:** 2024-07-16

**Authors:** Taiki Sunakawa, Shin Kobayashi, Masashi Kudo, Motokazu Sugimoto, Tatsushi Kobayashi, Naoto Gotohda

**Affiliations:** Department of Hepato-Biliary-Pancreatic Surgery, National Cancer Center Hospital East, 6-5-1 Kashiwanoha, Kashiwa, Chiba 277-8577, Japan; Advanced Clinical Research of Cancer, Juntendo University Graduate School of Medicine, 2-1-1, Hongo, Bunkyo-ku, Tokyo 113-8421, Japan; Department of Hepato-Biliary-Pancreatic Surgery, National Cancer Center Hospital East, 6-5-1 Kashiwanoha, Kashiwa, Chiba 277-8577, Japan; Department of Hepato-Biliary-Pancreatic Surgery, National Cancer Center Hospital East, 6-5-1 Kashiwanoha, Kashiwa, Chiba 277-8577, Japan; Department of Hepato-Biliary-Pancreatic Surgery, National Cancer Center Hospital East, 6-5-1 Kashiwanoha, Kashiwa, Chiba 277-8577, Japan; Department of Diagnostic Radiology, National Cancer Center Hospital East, 6-5-1 Kashiwanoha, Kashiwa, Chiba 277-8577, Japan; Department of Hepato-Biliary-Pancreatic Surgery, National Cancer Center Hospital East, 6-5-1 Kashiwanoha, Kashiwa, Chiba 277-8577, Japan; Advanced Clinical Research of Cancer, Juntendo University Graduate School of Medicine, 2-1-1, Hongo, Bunkyo-ku, Tokyo 113-8421, Japan

**Keywords:** paraganglioma, peripancreatic, pancreas-sparing tumor resection, urinary metanephrine, metaiodobenzylguanidine scintigraphy, case series

## Abstract

Paragangliomas (PGLs) located around the pancreas are rare and challenging to diagnose preoperatively. Tumor resection with pancreatectomy is often performed for peripancreatic PGL. However, pancreas-sparing tumor resection can be indicated with an accurate preoperative diagnosis. Six patients with pathologically diagnosed peripancreatic PGL were included. The clinical data were retrospectively collected from medical records. Five of them were suspected of peripancreatic PGL on imaging studies due to the fat plane identified between the tumor and pancreas, and subsequently diagnosed with PGL preoperatively based on elevated urinary catecholamine levels and/or metaiodobenzylguanidine scintigraphy without biopsy. All patients underwent pancreas-sparing tumor resection with negative surgical margins, and they did not develop postoperative complications related to potential damage to the pancreas. A fat plane between the tumor and pancreas on imaging studies and hormone levels are key findings for obtaining an accurate preoperative diagnosis of peripancreatic PGL, which can be managed with pancreas-sparing tumor resection.

## Introduction

Paragangliomas (PGLs) are rare neuroendocrine tumors originating from extra-adrenal chromaffin cells of the autonomic nervous system [1]. PGLs are commonly found in the head, neck and retroperitoneum. Further, they can be detected anywhere in the paraganglia. However, a retroperitoneal PGL around or within the pancreas is rare and often challenging to diagnose preoperatively (Zeng). Tumor resection is the standard treatment for PGLs, and tumor resection with pancreatectomy may be performed for peripancreatic PGL due to an equivocal preoperative diagnosis (Lanke). Pancreatic resection is associated with a risk of various complications such as pancreatic fistula, pseudoaneurysm rupture, cholangitis, and delayed gastric emptying [4]. Therefore, an accurate preoperative diagnosis of PGLs is essential to prevent pancreatectomy and associated complications for peripancreatic PGLs. The current case series aimed to present patients with peripancreatic PGL who underwent tumor resection successfully without pancreatectomy. Further, it elucidated crucial factors that should be considered when performing tumor resection without pancreatectomy for peripancreatic PGL.

## Case series

This case series included patients pathologically diagnosed with peripancreatic PGL resected at a hospital between 2002 and 2020. Peripancreatic PGL was defined as PGL that was in close contact with the pancreas on imaging examination. The clinical data of the patients were collected retrospectively from our medical records.

Six patients were histologically diagnosed with primary peripancreatic PGL based on surgical specimens. Table 1 shows the characteristics of all patients. Their mean age was 51 (standard deviation: ± 17) years. Two were men and four were women. Five patients had tumors around the pancreatic head, and one patient presented with a tumor around the pancreatic tail. The urinary catecholamine levels of five patients were measured. Results showed that all of them had elevated noradrenaline or normetanephrine levels. Hence, they were diagnosed with PGL preoperatively with diagnostic imaging followed by urinary catecholamine examination without tumor biopsy. In Case 3, the tumor was contiguous with the duodenum on imaging, and the patient was diagnosed with a duodenal gastrointestinal stromal tumor (GIST) preoperatively without urinary catecholamine measurement. A tumor biopsy was also not performed based on the clinical decision. All patients underwent tumor resection without pancreatic resection. However, two of them underwent simultaneous resection of other organs for different neoplasms. In Case 3, the tumor could be easily separated from the duodenum, and tumor resection was successfully performed. Two patients received preoperative alpha-adrenergic blockers to prevent severe blood pressure fluctuations during tumor resection. Five patients experienced intraoperative blood pressure fluctuations. Among them, two who did not receive antihypertensive treatment had severe blood pressure changes (>250 mmHg) due to tumor manipulation. None of the patients developed postoperative complications related to pancreatic injury. The median follow-up period was 105.5 (range, 2–180) months, and recurrence was not observed during this period. The two representative cases are shown in detail below.

**Table 1:** Clinicopathological features of six patients with peripancreatic paraganglioma.

No.	Age/sex	Clinical presentation	Imaging examinations	Location	Urine hormone test	EUS-FNA	Preoperative diagnosis	Surgical procedure	Intraoperative BP changes	Follow-up period (month)	Prognosis
1	36/female	Abdominal distension and sweating	CT, MRI, MIBG	Head	NAD ↑	No	PGL	Tumor resection	+	2	No recurrence
2	61/male	Incidental	CT, MRI, MIBG, PET	Head	NMN ↑	No	PGL + colon cancer	Tumor resection+ right hemicolectomy	+	24	No recurrence
3	51/female	Abdominal discomfort	CT, MRI	Head	NA	No	Duodenal GIST	Tumor resection	−	136	No recurrence
4	48/male	Incidental	CT, MRI, PET	Tail	NMN ↑	No	PGL	Tumor resection	+	119	No recurrence
5	71/female	Anorexia	CT, MRI, PET	Head	NMN ↑	No	PGL + gastric cancer	Tumor resection + distal gastrectomy	+	180	No recurrence
6	24/female	Vomiting	CT, MRI, MIBG	Head	NAD ↑	No	PGL	Tumor resection	−	92	No recurrence

EUS-FNA: endoscopic ultrasound-guided fine-needle aspiration, BP: blood pressure, CT: computed tomography, MRI: magnetic resonance imaging, MIBG: metaiodobenzylguanidine scintigraphy, PET: positron emission tomography, NMN: normetanephrine, NAD: noradrenaline, PGL: paraganglioma, GIST: gastrointestinal stromal tumor, NA: not available

### Case 1

**Figure 1:**
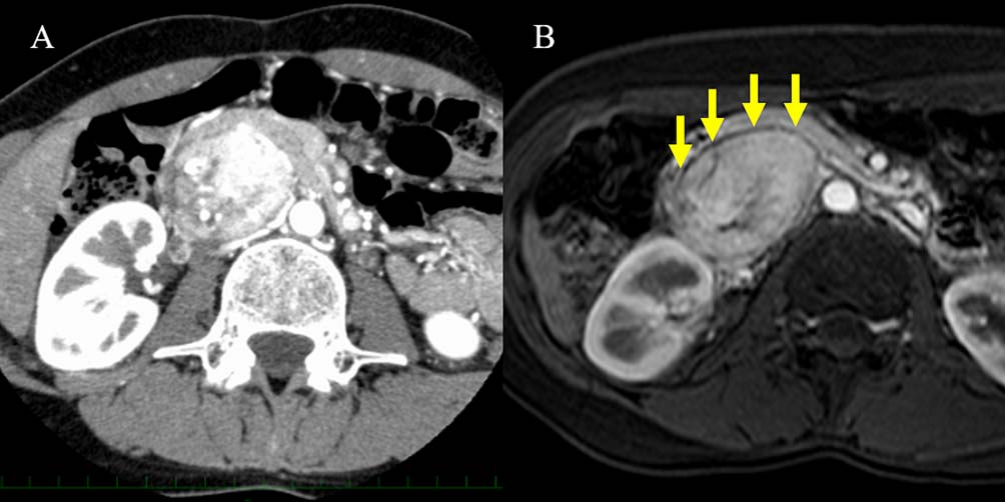
CT scan and MRI. (A) The hypervascular tumor was located dorsal to the pancreatic head in the arterial phase of contrast-enhanced CT scan. However, the outline of the tumor adjacent to the pancreas was somewhat unclear. (B) Fat-saturated T1-weighted MRI showing a clear plane between the tumor and pancreas.

A 36-year-old woman was referred to our hospital due to a complaint of abdominal distension with concomitant symptom of excessive sweating. Abdominal contrast-enhanced computed tomography (CT) scan showed a 7.3 × 5.3 cm^2^ hypervascular tumor located posterior to the pancreatic head in the arterial phase (Fig. 1A). There was no evidence of a continuous beak sign from the pancreatic parenchyma to the tumor wall. A fine discontinuity of the pancreas was more clearly observed on fat-saturated T1-weighted magnetic resonance imaging (MRI) than on CT scan (Fig. 1B). The patient’s urinary norepinephrine level was elevated; hence, she was diagnosed with PGL preoperatively. Alpha-adrenergic blockers were administered preoperatively to prevent blood pressure fluctuations. Then, the patient underwent tumor resection, and she had an uneventful course without postoperative complications.

### Case 2

**Figure 2:**
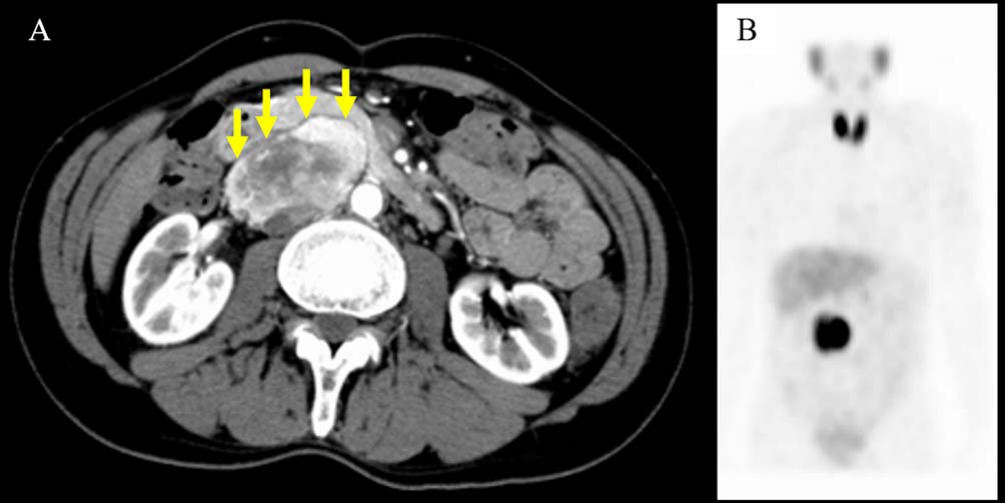
Computed tomography scan and metaiodobenzylguanidine scintigraphy. (A) Imaging findings showing a well-defined mass with heterogeneous enhancement located adjacent to the pancreatic head. A fat plane was identified between the mass and pancreas (arrows). (B) Metaiodobenzylguanidine scintigraphy revealed accumulation at the same site of the abdominal tumor.

**Figure 3:**
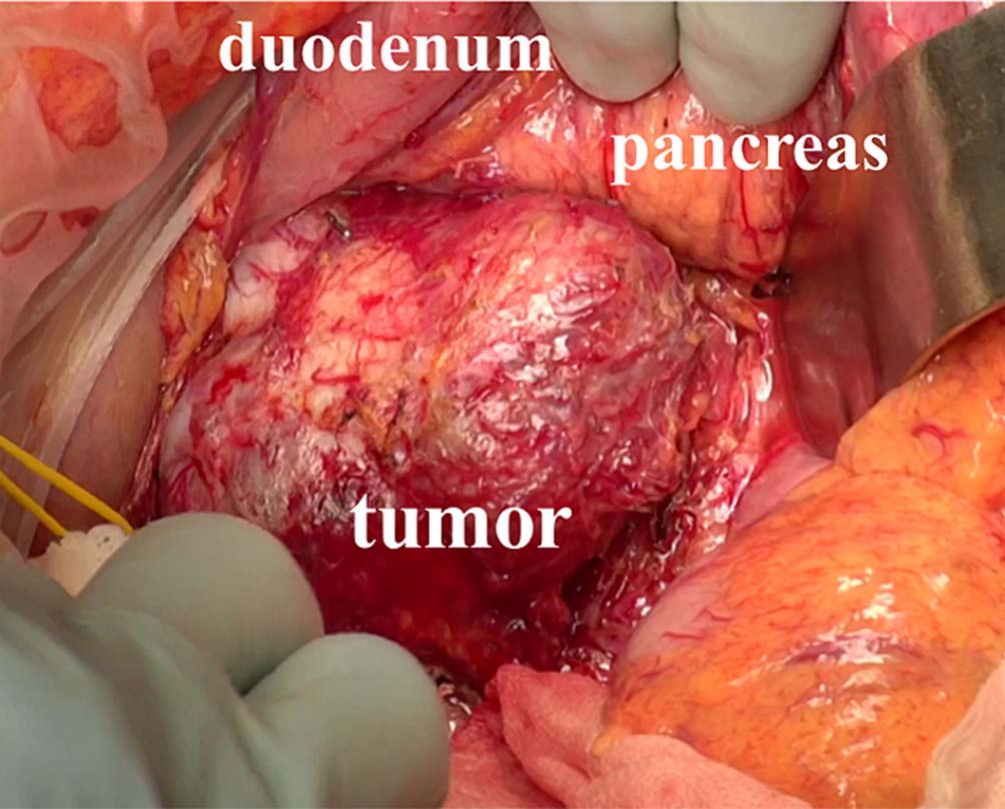
Intraoperative findings. The tumor was located behind the pancreas and the duodenum, and the mass was easy to separate from the surrounding organs.

**Figure 4:**
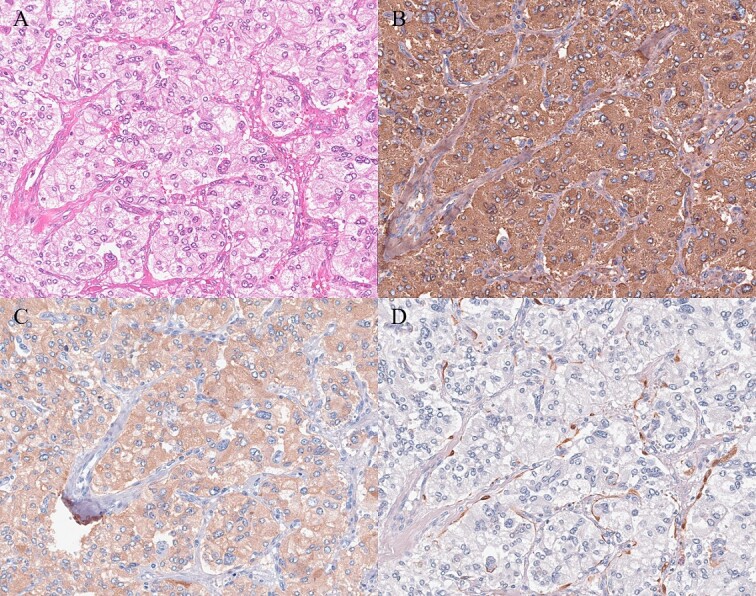
Histopathologic and immunohistochemical findings. (A) Hematoxylin and eosin staining revealed tumor cells with round nuclei and eosinophilic cytoplasm arranged in a typical zellballen pattern (200×). (B, C) Immunohistochemistry showed positive staining (B chromogranin, 200×; C synaptophysin, 200×). (D) Immunohistochemical staining for S-100 revealed the presence of sustentacular spindle cells (200×).

A 61-year-old man underwent a positive fecal occult blood test and was diagnosed with adenocarcinoma of the transverse colon based on colonoscopy and biopsy results. During the preoperative evaluation, a hypervascular neoplasm located posterior to the pancreatic head was incidentally detected on abdominal contrast-enhanced CT scan (Fig. 2A). The arterial phase of the contrast-enhanced CT scan showed a 6.5 × 3.5 cm^2^ heterogeneous mass with fat plane between the mass and pancreas while a beak sign was not observed. The patient’s medical history included hypertension, and PGL was suspected based on CT scan images and medical history. His laboratory data were unremarkable except for high urinary metanephrine levels. Metaiodobenzylguanidine (MIBG) scintigraphy was also performed and showed an abnormal uptake at the site of the neoplasm (Fig. 2B). Thus, the patient was preoperatively diagnosed with PGL. According to the intraoperative findings, combined resection with pancreatectomy was unnecessary (Fig. 3). Hence, he underwent tumor resection for PGL and right hemicolectomy for transverse colon cancer. Histopathological and immunohistochemical findings confirmed the diagnosis of PGL (Fig. 4). The patient had an uneventful course except for a mild superficial surgical site infection (Clavien–Dindo grade I complication), and no tumor recurrence was observed for 24 months.

## Discussion

We reported six patients who underwent pancreas-sparing tumor resection for peripancreatic PGL. Five patients were diagnosed with PGL preoperatively by diagnostic imaging followed by elevated urinary catecholamine levels without tumor biopsy. Meanwhile, one patient was preoperatively diagnosed with duodenal GIST without urinalysis. Imaging of the extra-pancreatic tumor, which shows the presence of a fat plane between the mass and the pancreas and the absence of a beak sign, followed by a comprehensive work-up, including plasma, or urinary catecholamine level measurement and MIBG scintigraphy, may help surgeons obtain an accurate preoperative diagnosis without tumor biopsy that may be otherwise associated with a risk of bleeding due to hypervascular nature of the tumor. An accurate preoperative diagnosis may enable surgeons to plan pancreas-sparing resection for peripancreatic PGLs with appropriate perioperative blood pressure management using alpha-adrenergic blockers.

The differential diagnoses of a hypervascular tumor in and around the pancreas include pancreatic neuroendocrine tumor (pNET), GIST and solid pseudopapillary neoplasm. Peripancreatic PGL and pNET are more likely to have similar radiologic findings. Thus, they are often challenging to distinguish. In a previous report, the identification of a fat plane between the mass and the adjacent pancreas may indicate PGL [5]. The beak sign and the feeding arteries are also useful in assessing the origin of the tumor organ [6]. Therefore, imaging findings that are indicative of an extra-pancreatic lesion such as the presence of a fat plane or the absence of a beak sign are important in considering peripancreatic PGL as a differential diagnosis. As shown in Case 1, fat-saturated MRI may be more effective in detecting a fat plane than CT scan in some patients. If imaging findings indicate that the tumor is extrapancreatic, a comprehensive work-up can be useful in obtaining an accurate preoperative diagnosis of peripancreatic PGL. The urinary or plasma levels of catecholamines and their metabolites are used to diagnose PGL [7]. In a previous report, 66.6% of patients with PGL had elevated urinary norepinephrine levels [8]. A previous report showed that the combined sensitivity based on three catecholamine studies was 89.9%, and the false-negative rate was 14.2% [9]. Among our patients, five underwent the 24-hour urine test, and all of them had elevated urinary normetanephrine or norepinephrine levels. Therefore, catecholamine levels should be measured if PGL is suspected on imaging studies. In addition, MIBG scintigraphy can be performed to validate the preoperative diagnosis based on the laboratory tests and to search for multicentric lesions [9].

Perioperative management is required to facilitate a safe treatment and achieve favorable outcomes. In preoperative medical management, alpha-adrenergic blocker is recommended to prevent perioperative hemodynamic instability [10, 11]. The clinical guideline recommends that medical treatment should be started at least 7 days preoperatively to stabilize blood pressure [10]. In addition, a high-sodium diet and fluid intake should be implemented to prevent severe hypotension after tumor removal. In our study, five patients experienced intraoperative blood pressure fluctuation, including two with severe hypertension due to inadequate preoperative medical preparation. However, all surgeries were successfully performed. A previous report showed one case in which surgery was discontinued due to severe intraoperative hypertension [3]. Taken together, pancreas-sparing tumor resection for peripancreatic PGL can be safely performed based on an accurate preoperative diagnosis and proper perioperative management.

There are approximately 50 patients diagnosed with pancreatic or peripancreatic PGL in the literature. Among them, 10 were accurately diagnosed preoperatively. Table 2 shows 15 patients diagnosed with PGL preoperatively, including 10 previously published cases and ours [2, 3, 11–15]. In addition to our patients, six patients received surgical treatment, and at least one patient underwent tumor resection. However, data on the surgical procedure were not available. A preoperative diagnosis of peripancreatic PGL may lead to planned pancreas-sparing tumor resection. Pancreatectomy is frequently associated with postoperative complications such as pancreatic fistula, pseudoaneurysm rupture, cholangitis and delayed gastric emptying [4]. In our opinion, an accurate preoperative diagnosis is important for preventing these complications. In contrast, eight patients were diagnosed based on endoscopic ultrasound-guided fine-needle aspiration (EUS-FNA) findings. However, EUS-FNA is associated with a risk of bleeding especially in cases with hypervascular tumor such as PGL, and it may misdiagnose peripancreatic PGL with pNET as both conditions have similar morphologic characteristics on cytology. Moreover, the usefulness of EUS-FNA in diagnosing peripancreatic PGL is controversial [2, 11]. Some guidelines recommend that the initial biochemical tests such as plasma-free metanephrine or urinary fractionated metanephrine levels should be performed to diagnose PGL [10]. Our patients’ preoperative diagnosis was made via imaging study and subsequent biochemical tests without EUS-FNA. Therefore, in cases with suspected peripancreatic PGL based on imaging findings, measurement of plasma or urinary catecholamines should be performed first as it is easier and less invasive, and EUS-FNA should be avoided as much as possible due to its potential risk of bleeding.

**Table 2:** Accurate diagnosis before resection and medical examinations for pancreatic/peripancreatic paraganglioma.

First author	Age/sex	Urinary catecholamines test	MIBG scintigraphy	EUS-FNA	Preoperative diagnosis	Treatment
Zhang L [2]	63/female	Normal	Uptake (+)	Yes	PGL	Surgical resection (details unknown)
Zhang L [2]	50/female	No	No	Yes	PGL	Chemotherapy
Lanke G [3]	73/female	NA	No	Yes	PGL	Follow-up
Endo Y [11]	22/male	NMN ↑	Uptake (+)	No	PGL	Surgical resection (details unknown)
Thakur A [12]	58/male	No	No	Yes	PGL	Internal radiotherapy
Perrot G [13]	41/female	NMN ↑	Uptake (+)	EUS only	PGL	Tumor resection
Nguyen E [14]	70/female	No	No	Yes	PGL	Surgical resection (details unknown)
Singhi AD [15]	52/female	NA	No	Yes	PGL	Chemoradiation therapy
Singhi AD [15]	54/female	NA	No	Yes	PGL	Surgical resection (details unknown)
Singhi AD [15]	44/male	NA	No	Yes	PGL	Surgical resection (details unknown)
Present Case 1	61/male	NMN ↑	Uptake (+)	No	PGL	Tumor resection
Present Case 2	36/female	NAD ↑	Uptake (+)	No	PGL	Tumor resection
Present Case 4	48/male	NMN ↑	No	No	PGL	Tumor resection
Present Case 5	71/female	NMN ↑	No	No	PGL	Tumor resection
Present Case 6	24/female	NAD ↑	Uptake (+)	No	PGL	Tumor resection

MIBG: metaiodobenzylguanidine, EUS-FNA: endoscopic ultrasound-guided fine needle aspiration, NA: not available, NMN: normetanephrine, NAD: noradrenaline, PGL: paraganglioma

The study had several limitations, including a small number of cases and a retrospective single center design. However, in all cases, the tumor was resected without pancreatectomy, which emphasizes the importance of an accurate preoperative diagnosis.

Cautious observation of imaging findings such as a fat plane and beak sign, followed by biochemical tests for catecholamine levels or MIBG scintigraphy, may help obtain an accurate preoperative diagnosis of peripancreatic PGL without tumor biopsy and facilitate pancreas-sparing resection with appropriate perioperative blood pressure management. PGL should be included in the differential diagnosis of hypervascular neoplasm in the peripancreatic area.

## Data Availability

The datasets used and analyzed in this study are available from the corresponding authors upon reasonable request.
